# Supplementary material for: Plasma Endocan as a Biomarker of Thrombotic Events in COVID-19 Patients
Source: J Clin Med. 2022 Sep 22;11(19):5560. doi: 10.3390/jcm11195560 (PMC9572304; doi:10.3390/jcm11195560)
Supplement: Supplementary file 1 [file jcm-11-05560-s001.zip › jcm-1900159-supplementary.pdf]

Table S1. List of patients with demographics, disease severity, comorbidities and therapies.

ID_Patient	*Gender*	Age (Years)	COVID-19 Severity	Thrombotic event (TE) (yes/no)	if yes, type of TE	Cardiovascular disease (yes/no)	Overweight /obesity (yes/no)	Hypertension (yes/no)	Diabetes (yes/no)	Chronic respiratory failure (yes/no)	Systemic AI disease (yes/no)	antiviral treatment (yes/no)	if yes, specify antiviral treatment	Immunomodulator treatment (yes/no)	if yes, specify immunomodulator treatment
ID065	*Man*	50	stage 1	yes	Thrombosis on material	no	no	no	no	no	no	yes	Kaletra	no	
ID107	*Man*	66	stage 3	yes	Jugular thrombosis	yes	yes	yes	yes	no	no	yes	HCQ	yes	Sarilumab
ID113	*Man*	50	stage 3	yes	Jugular thrombosis	no	yes	no	no	no	no	no		no	
ID114	*Man*	56.2	stage 3	yes	Deep veinous thrombosis	yes	yes	yes	no	no	no	yes	Kaletra	yes	Sarilumab
ID126	*Man*	37.3	stage 3	yes	Jugular thrombosis	no	yes	no	no	no	no	yes	Remdesivir	no	
ID011	*Man*	45	stage 3	yes	PE	yes	no	no	no	no	no	no		no	
ID015	*Woman*	77	stage 3	yes	PE	no	no	no	no	no	no	no		no	
ID024	*Man*	50.8	stage 3	yes	PE	yes	no	no	no	no	no	no		no	
ID026	*Man*	52	stage 3	yes	PE	yes	yes	yes	no	no	no	no		no	
ID052	*Man*	77.7	stage 3	yes	PE	no	no	no	no	no	no	yes	Remdesivir	no	
ID100	*Woman*	26.8	stage 3	yes	PE	no	yes	yes	no	no	no	no		yes	Anakinra
ID122	*Woman*	61	stage 2	yes	PE	no	yes	no	yes	no	no	yes	Kaletra	yes	Sarilumab
ID156	*Man*	50	stage 3	yes	PE	no	yes	no	no	no	no	no		no	
ID160	*Man*	60	stage 1	yes	PE	no	yes	no	no	no	no	no		no	
ID171	*Man*	65	stage 2	yes	PE	yes	yes	yes	no	no	no	yes	Kaletra	no	
ID183	*Man*	71.6	stage 1	yes	PE	no	no	no	no	no	no	yes	Kaletra	no	
ID005	*Man*	50	stage 0	no		no	no	no	no	no	no	no		no	
ID006	*Woman*	55	stage 3	no		yes	yes	yes	yes	no	no	no		no	
ID008	*Man*	72	stage 3	no		yes	yes	yes	no	no	no	no		no	
ID012	*Man*	35	stage 0	no		no	no	no	no	no	no	no		no	
ID018	*Woman*	47.4	stage 0	no		no	no	no	no	no	no	no		no	
ID020	*Woman*	76.1	stage 1	no		no	no	no	no	no	no	no		no	
ID021	*Man*	62.5	stage 3	no		yes	yes	yes	no	no	no	no		no	

ID028	*Woman*	42.7	stage 0	no	no	no	no	no	no	no	no		no	
ID029	*Man*	55.3	stage 0	no	no	yes	no	no	no	no	no		no	
ID033	*Woman*	75.2	stage 1	no	yes	yes	yes	yes	no	no	no		no	
ID034	*Woman*	46	stage 0	no	no	no	no	no	no	no	no		no	
ID039	*Woman*	27.3	stage 0	no	no	no	no	no	no	no	no		no	
ID040	*Woman*	84	stage 3	no	yes	no	no	yes	yes	yes	yes	HCQ	3	
ID043	*Man*	69.1	stage 3	no	no	yes	no	no	no	no	no		no	
ID044	*Woman*	53.3	stage 2	no	no	no	no	no	no	no	no		yes	Anakinra
ID045	*Man*	43.4	stage 0	no	no	no	no	no	no	no	no		no	
ID046	*Woman*	69	stage 1	no	yes	yes	no	no	no	no	no		no	
ID049	*Man*	66	stage 2	no	yes	no	no	yes	no	no	no		yes	Sarilumab
ID051	*Man*	54	stage 3	no	no	yes	no	no	no	no	no		no	
ID054	*Man*	94.4	stage 0	no	yes	no	no	no	no	no	no		no	
ID057	*Man*	77.5	stage 1	no	yes	yes	yes	yes	no	no	yes	Kaletra	no	
ID058	*Man*	40.6	stage 1	no	no	yes	no	no	no	no	no		yes	Sarilumab
ID059	*Man*	45	stage 3	no	no	yes	no	no	no	no	yes	HCQ	no	
ID060	*Woman*	57.9	stage 0	no	no	no	no	no	no	no	no		no	
ID064	*Woman*	69.4	stage 2	no	no	no	no	no	yes	no	no		no	
ID069	*Woman*	80.3	stage 1	no	yes	no	yes	yes	no	no	no		no	
ID070	*Woman*	80.3	stage 1	no	yes	no	yes	yes	no	no	no		no	
ID071	*Man*	76.3	stage 2	no	yes	no	yes	no	no	no	yes	Kaletra	no	
ID072	*Man*	64.8	stage 1	no	no	no	no	no	no	no	no		no	
ID073	*Woman*	62	stage 1	no	yes	no	no	no	no	yes	yes	Kaletra	yes	IFN
ID077	*Woman*	21	stage 0	no	no	no	no	no	no	no	no		no	
ID085	*Man*	34	stage 1	no	no	no	no	no	no	no	no		no	
ID087	*Man*	64.2	stage 3	no	no	yes	no	no	no	no	yes	HCQ + azithromy-cine	no	
ID089	*Woman*	70	stage 1	no	no	no	no	no	no	yes	yes	Kaletra	no	
ID090	*Man*	74.2	stage 1	no	yes	no	yes	no	yes	no	no		no	
ID091	*Woman*	82	stage 3	no	yes	no	yes	no	no	no	yes	Kaletra	no	
ID095	*Woman*	21.3	stage 0	no	no	no	no	no	no	no	no		no	
ID097	*Woman*	58	stage 2	no	no	yes	no	yes	no	no	yes	HCQ + azithromy-cine	no	
ID103	*Woman*	33.4	stage 0	no	no	no	no	no	no	no	no		no	
ID106	*Woman*	88.2	stage 2	no	yes	no	no	no	no	no	yes	Kaletra	no	
ID110	*Man*	61.9	stage 3	no	no	yes	no	yes	no	no	yes	Remdesivir	yes	Anakinra

ID112	*Woman*	52	stage 2	no	no	yes	no	no	no	no	yes	Kaletra	no	
ID121	*Woman*	64.3	stage 2	no	yes	yes	yes	yes	no	no	no		no	
ID125	*Man*	57	stage 2	no	no	no	no	no	no	no	yes	Kaletra	yes	Sarilumab
ID128	*Man*	73	stage 3	no	yes	yes	yes	yes	no	no	no		no	
ID129	*Man*	77.2	stage 0	no	yes	yes	yes	no	no	no	no		no	
ID130	*Woman*	82	stage 2	no	no	no	no	no	no	no	yes	Kaletra	no	
ID134	*Woman*	66.1	stage 2	no	yes	yes	yes	no	no	no	no		no	
ID135	*Woman*	37.3	stage 0	no	no	no	no	no	no	no	no		no	
ID142	*Man*	82.2	stage 3	no	yes	no	yes	no	yes	no	no		yes	Anakinra
ID144	*Man*	50	stage 1	no	no	no	no	no	no	no	no		yes	Sarilumab
ID145	*Woman*	64.3	stage 3	no	no	yes	no	yes	no	no	no		no	
ID147	*Man*	62.4	stage 1	no	yes	yes	yes	no	no	no	yes	Kaletra	no	
ID158	*Man*	38	stage 1	no	no	no	no	no	no	no	no		no	
ID161	*Man*	72.2	stage 2	no	yes	yes	yes	no	no	no	no		no	
ID163	*Man*	49	stage 1	no	no	no	no	no	no	no	no		no	
ID166	*Man*	73	stage 1	no	yes	no	yes	yes	no	no	no		no	
ID170	*Woman*	57.2	stage 0	no	no	no	no	no	no	no	no		no	
ID172	*Man*	62	stage 2	no	yes	no	yes	yes	no	no	no		no	
ID175	*Woman*	60	stage 1	no	yes	yes	no	yes	no	no	yes	Kaletra	no	
ID180	*Woman*	31.5	stage 0	no	no	no	no	no	no	no	no		no	
ID185	*Woman*	32	stage 0	no	no	no	no	no	no	no	no		no	
ID188	*Woman*	45	stage 2	no	no	yes	no	no	no	no	yes	Kaletra	yes	Sarilumab
